# Survey dataset on factors that influence satisfaction of clients with architectural services in Lagos State, Nigeria

**DOI:** 10.1016/j.dib.2018.07.055

**Published:** 2018-07-29

**Authors:** Adedapo Oluwatayo, Adedotun O. Akinola, Ikenna U. Agomuo, Stephanie O. Mozimo, Chiekwugo C. Onwuka, Hilary I. Okagbue

**Affiliations:** a Department of Architecture, Covenant University, Ota, Nigeria; b Department of Mathematics, Covenant University, Ota, Nigeria

**Keywords:** Architectural services, Client satisfaction, Statistics, Satisfaction, Nigeria

## Abstract

The retention of clients of professional service practitioners is often dependent on their satisfaction with the services they obtain. This article presents data on the factors that influence the satisfaction of clients with architectural services in Lagos State, Nigeria. Data were obtained from a cross-sectional survey research, which adopted random sampling of clients from six estates in the State. The questionnaire was used as an instrument for the survey. The response rate was 81.3%. The dataset is made available in tables and charts of frequencies and means. The data are of interest to researchers in the professional service organisation, management and those in the decision sciences. The data could also be of interest to practitioners who may further analyse the data to develop business strategies to satisfy their clients.

## Specifications Table

**Table:** 

Subject area	*Architecture and construction*
More specific subject area	*Architectural professional practice*
Type of data	*Tables and figures*
How data was acquired	*Field survey using questionnaires*
Data format	*Raw and analysed*
Experimental factors	*Cross-sectional survey of clients who have procured architectural services*.
Experimental features	*Random sampling, frequencies, Mean ranking*
Data source location	*Lagos, Nigeria*
Data accessibility	*All the data are included in this data article*

## Value of the data

• The data presents indications of the factors that influence the satisfaction of clients with architectural services in Lagos, Nigeria [1].
• The data can be helpful to practitioners in developing business strategies to satisfy their clients [2].
• The data can be helpful to researchers in the fields of professional service management and those in the decision sciences to develop theories of professional service client satisfaction [3].
• The dataset can be useful to architectural professional bodies in determining areas where architects are deficient in satisfying their clients, and thus organising professional development programmes for building the capacities of their members in those areas [4].

## 1 Data

**Fig. 1:**
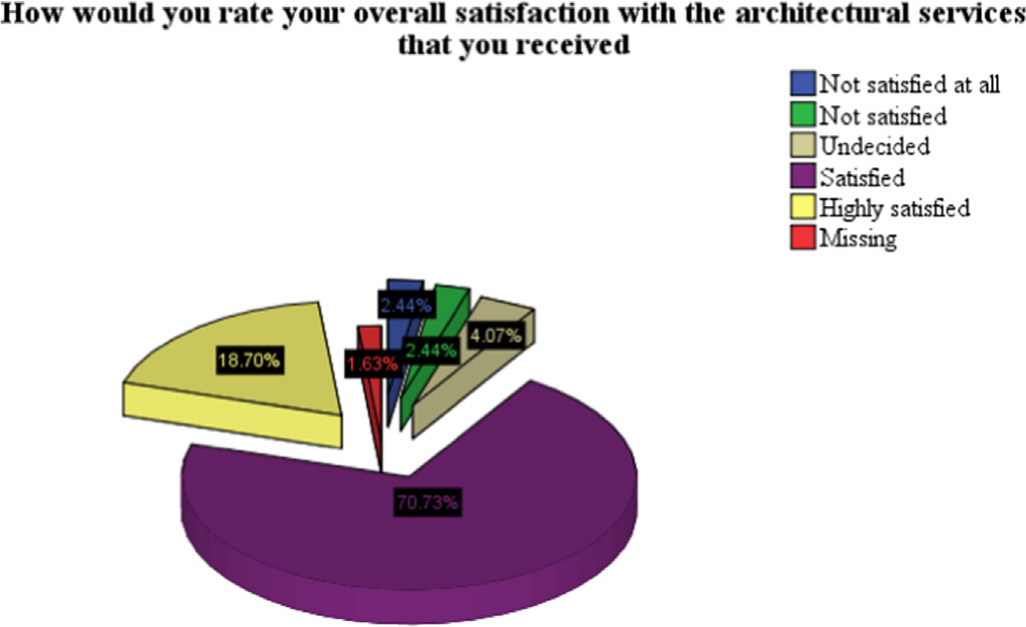
Overall satisfaction with architectural services.

The dataset contains empirical evidence of the factors that influence satisfaction of clients with architectural services in Lagos State, Nigeria A total of 150 questionnaires were administered at random to various clients who have procured architectural services, 122 of which were filled and returned. Table 1 shows the socioeconomic characteristics of respondents, while Table 2 shows the response rate per estate. The summary of the data presented in Tables 3 and 4 are for the types of services the clients obtained from the architect and the mean ranking of the criteria used in selecting those architects respectively. Fig. 1 presents the date on the level of satisfaction of the clients. The data on satisfaction is presented in Table 5, which presents the mean ranking of the level of satisfaction of the clients with different aspects of the services procured (Table 6).

**Table 1:** Socio economic characteristics of respondents.

**Characteristics of respondents**		**Percent**
**Gender of respondents**	Male	23.58
	Female	76.42
**Age in years**	Below 30 years	20.33
	31–39	20.33
	40–49	29.26
	50 and above	30.08
**Marital status**	Single	17.21
	Married	70.49
	Widowed	8.20
	Separated	2.45
	Divorced	1.64
**Highest education qualification of respondents**	No response	0.8
	No schooling	1.6
	Primary education	1.6
	Secondary education	2.5
	OND	2.5
	HND	12.3
	Bachelor׳s degree	40.2
	Masters Degree	36.0
	Others	2.5
**Occupation of respondents**	No response	0.80
	Employed for wages	42.28
	Self employed	42.28
	Out of work and looking for work	1.63
	A home maker	0.81
	A student	2.44
	Military	2.44
	Retired	7.32
**Average monthly income**	N50,000 or less	4.06
	N 50,001–N150,000	17.89
	N150,001–N250,000	21.14
	N250,001–N350,000	14.63
	Above N350,000	42.28

**Table 2:** Number of administered and valid questionnaires.

	Number of questionnaires administered	Number of questionnaires returned
Estate 1 (Victoria Island, Lagos State)	25	20
Estate 2 (Amuwo Odofin Lagos State)	25	25
Estate 3 (Lekki, Lagos State)	25	20
Estate 4 (Idimu Lagos)	25	17
Estate 5 (Lekki, Lagos State)	25	20
Estate 6 (Surulere, Lagos State)	25	20
Total	150	122

**Table 3:** Types of services architect was commissioned for.

	Yes	No
personal house	60.8%	40.2%
educational building	25.8%	75.4%
rental apartment	18.3%	82.8%
industrial building	10.8%	90.2%
healthcare building	7.5%	93.4%
rental apartment	4.2%	95.9%
religious building	2.5%	98.4%
entertainment building	1.7%	99.2%

**Table 4:** Criteria used by clients for the selection of architects.

	*N*	Mean	Std. Deviation
Ease of communication	119	4.29	3.856
Experience	122	4.11	0.880
Availability	118	4.03	1.194
Service reliability	120	3.91	0.870
Competence/professionalism	121	3.89	1.055
Professional advice	120	3.78	0.945
Expertise in design of particular building types	122	3.75	1.070
Recommendation	121	3.74	1.006
Convenience	119	3.73	0.909
Quality of previous service	122	3.71	1.040
Reputation	122	3.68	1.014
Friendliness	121	3.66	0.954
Value added services	121	3.64	0.965
Client service	119	3.64	1.006
Accessibility of architect in urgency	122	3.57	1.143
Patience and help established relationship	121	3.54	1.103
IT proficiency	122	3.48	0.947
Financial Consideration	118	3.45	1.099
Personal Relationship	120	3.33	1.252
Past relationship	120	3.31	1.282
Geographical location	121	3.28	1.149
International scope of architect	120	3.25	1.386
Religious affinity	122	2.61	1.256
Ethnic affinity	118	2.31	1.182

**Table 5:** Respondents’ satisfaction with the architectural services.

	*N*	Mean	Std. Deviation
Attainment of design requirement	122	4.04	0.697
Effective communication	122	4.02	0.766
Adequate consultant experience	122	3.98	0.765
Display of expertise	122	3.93	0.689
Speed of service	122	3.90	0.754
Proper methods in rendering service	122	3.89	0.695
Decision making	122	3.88	0.819
Proper coordination of resources	122	3.86	0.826
Construction and supervision	121	3.79	0.939
Effective control of budget	121	3.73	0.827
Cost estimates	122	3.72	0.816
Labour productivity	121	3.72	0.788
Waste reduction/ management	120	3.60	0.929

**Table 6:** Factors that influence clients’ satisfaction with architectural services.

	*N*	Mean	Std. Deviation
The architect displayed adequate knowledge about architecture	121	4.16	0.730
The architect understood the kind of help I wanted	121	4.16	0.837
The architect was friendly	119	4.06	0.784
The architect understood my specific needs	119	4.05	0.832
I received the type of service I was looking for	121	4.05	0.773
The architect provided easy access to needed information	120	4.04	0.782
The architect displayed competence	120	4.03	0.788
The architect rendered quality service.	120	4.02	0.778
The architect always answered my questions satisfactorily	120	4.02	0.879
The architect׳s office was welcoming	120	3.99	0.884
The architect was dependable in handling service problems	121	3.98	0.671
I like the way the architect relates with me	121	3.98	0.846
The architect maintained professionalism	119	3.98	0.802
The architect was consistently courteous	117	3.98	0.799
The architect was always willing to help	119	3.97	0.736
The architect informed me about decisions made on my behalf	120	3.96	0.679
The architect explained the process well	119	3.95	0.735
The architect always properly handled problems that arose during the course of the project	121	3.92	0.881
The architect met my expectations	119	3.92	0.829
The architect was caring and concerned	120	3.89	0.924
The architect provided services at promised time	118	3.88	0.879
The architect is always available when I want to discuss	121	3.87	0.865
The architect was prompt at attending to my requests	120	3.87	0.829
The architect kept my dealings confidential	120	3.86	1.031
The architect follows through on his promises	120	3.81	1.095
The architect gave me personal attention	120	3.75	1.094
The architect did things right the first time	119	3.75	1.019
The charges were reasonable	120	3.66	1.104
The architect seemed to have a different idea about my project objective	121	3.34	0.962
The architect was often too busy to attend to my requests	118	3.28	1.226

**Fig. 2:**
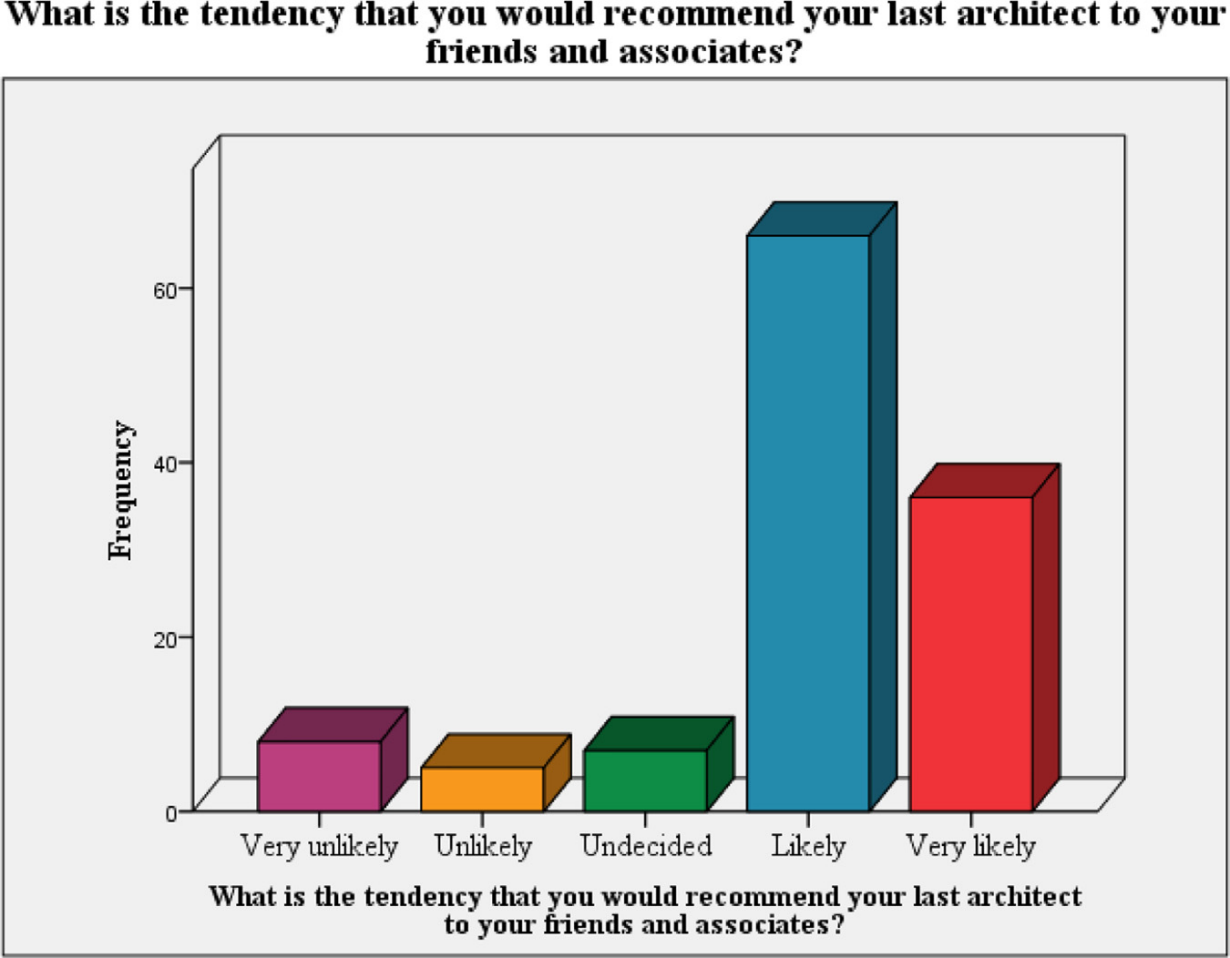
Tendency to recommend last architect to friends and associates.

**Fig. 3:**
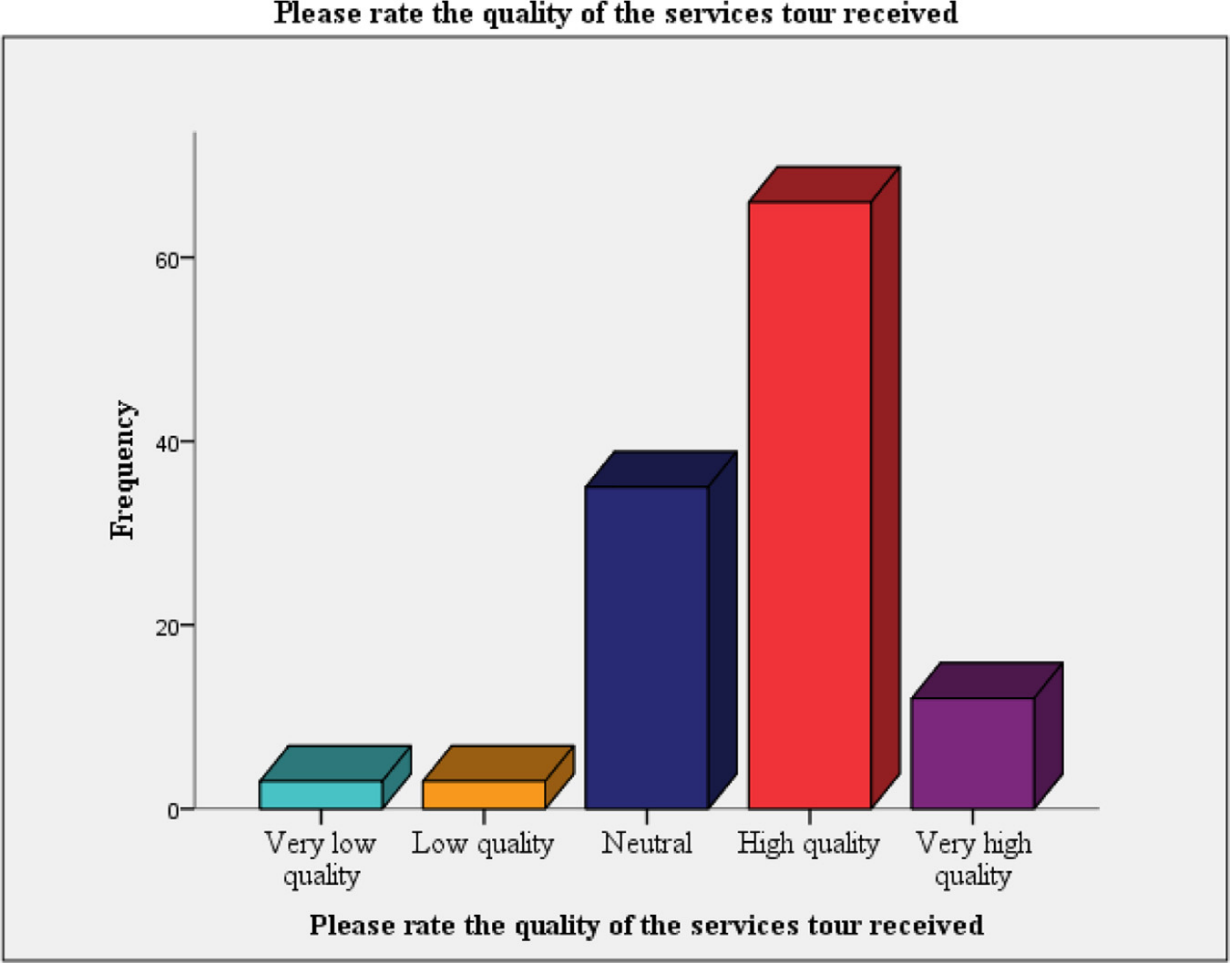
The quality of the services that the clients received.

**Fig. 4:**
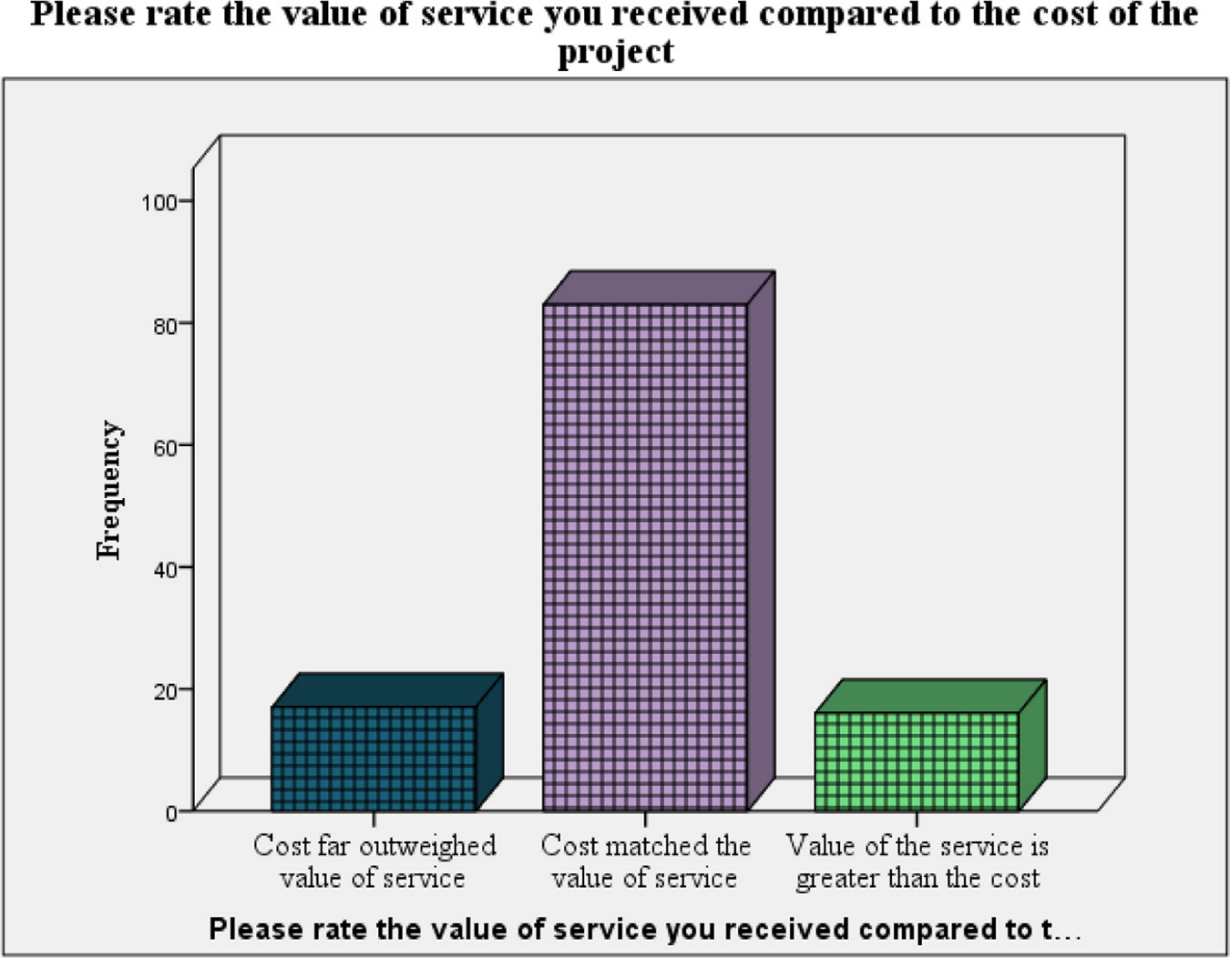
The value of services the clients received compared to the cost of the project.

The data on assessment of the clients on propensity to recommend the architect to other was presented in Fig. 2. Figs. 3 and 4 present the data on the assessment of the quality of the service and the cost versus quality assessment respectively. The questionnaire can be assessed as Supplementary data 1 while the raw data can be assessed as Supplementary data 2.

Fig. 4 below shows our respondents’ rating of the quality of services received. Majority of the respondents received high quality of service.

## 2 Experimental design, materials and methods

### 2.1 Sample and data collection

The data for this study were obtained from building owners who had procured the services of architects in designing and constructing their buildings. For this purpose, estates in different locations in Lagos, Nigeria were purposefully selected. Lagos is a place that is considered the commercial centre of Nigeria and the rate of urbanisation in the state is higher than that of other states. Similarly, the level of construction projects in the state is also higher than in other states. The sample consisted of building owners in the selected estates. The first task for the researchers was to locate these building owners, and then ascertain if they sought for architectural consultancy in their building projects. Once this was confirmed, the respondents were asked to fill the questionnaire. The data collected were analysed using SPSS version 21. Data were analysed using descriptive and statistical tools, some of which can be seen in [5], [6], [7], [8], [9], [10], [11], [12], [13], [14], [15], [16], [17], [18], [19], [20], [21], [22], [23], [24].

### 2.2 Implications of study

The data adds to knowledge by providing empirical data on the satisfaction of clients with architectural services from the context of Lagos State, Nigeria. The data serve as a standard to ascertain the level of satisfaction of clients of architects. It will also serve as a basis for further studies in other locations. The data suggest areas that clients are not so satisfied with their architect and the key factors to be addressed to client satisfaction.
