# Supplementary material for: Survey dataset on factors that influence satisfaction of clients with architectural services in Lagos State, Nigeria
Source: Data Brief. 2018 Jul 29;20:118–25. doi: 10.1016/j.dib.2018.07.055 (PMC6088564; doi:10.1016/j.dib.2018.07.055)
Supplement: Supplementary file 1 — Supplementary material. [file mmc1.pdf]

COVENANT UNIVERSITY
COLLEGE OF SCIENCE AND TECHNOLOGY
DEPARTMENT OF MATHEMATICS

CANAANLAND, KM 10, IDIROKO ROAD
P.M.B 1023, OTA, OGUN STATE, NIGERIA
www.covenantuniversity.edu.ng, mat.covenantuniversity.edu.ng

EXTERNAL MEMO

To: Editor, Data in Brief
From: Corresponding Author
Date: 10th July, 2018
Subject: **Conflict of Interest**

I thereby declare the absence of any conflict of interest among the authors.

The authors have read the final draft and unanimously agreed that the paper be sent for review.

Hilary I. Okagbue
Department of Mathematics, College of Science and Technology, Covenant University, Ota.
Google Scholar: *Hilary Izuchukwu Okagbue*
Research Gate: *Hilary Okagbue*
Live DNA: 234.14453
Scopus ID: 56438006100
ORCID: 0000-0002-3779-9763
