# Supplementary material for: Survey dataset on factors that influence satisfaction of clients with architectural services in Lagos State, Nigeria
Source: Data Brief. 2018 Jul 29;20:118–25. doi: 10.1016/j.dib.2018.07.055 (PMC6088564; doi:10.1016/j.dib.2018.07.055)
Supplement: Supplementary file 2 — Supplementary material. [file mmc2.docx]

**APPENDIX**

**QUESTIONNAIRE**

**
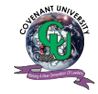
**

**COLLEGE OF SCIENCE AND TECHNOLOGY**

**DEPARTMENT OF ARCHITECTURE**

Dear Sir/ Madam

**FACTORS THAT INFLUENCE SATISFACTION OF CLIENTS WITH ARCHITECTURAL SERVICES**

Kindly give candid answers to the questions below. The questionnaire is designed to collect information for our research on “Factors that Influence Satisfaction of Clients with Architectural Services”. Please be assured that the information provided by you

**SECTION A**

**SOCIO- DEMOGRAPHIC CHARACTERISTICS OF THE RESPONDENT**

***INSTRUCTION:*** *Please provide appropriate answers by ticking (√) the option that best agrees with your opinion.*

1. Gender: Male [ ]^1^ Female [ ] ^2^
2. Age Group: Below 30 years [ ]^1^ 30 – 39 years [ ]^2^  40 – 49year s [ ]^3^ Over 50 years [ ]^4^
3. Marital Status: Single [ ] Married [ ] Widowed [ ] Divorced [ ] Separated [ ]
4. Highest Educational Qualification: No schooling completed [ ] Primary Education [ ] Secondary school [ ] OND [ ]^1^ HND [ ]^2^ Bachelor’s degree [ ] Master’s degree [ ] Others (please specify)^5^.......................
5. Occupation: Employed for wages [ ] Self-employed [ ] Out of work and looking for work [ ]

Out of work but not currently looking for work [ ] A homemaker [ ] A student [ ] Military [ ] Retired [ ] Unable to work [ ]

1. What is your average monthly income? N50,000 or less [ ] N50,001-N150,000 [ ] N150,001- N250,000 [ ] N250,001- N350,000 [ ] Above N350,000 [ ]
2. On the average, how many architects would you say you know?

Only 1 [ ] 2-5 [ ] 5-10[ ] More than10 [ ]

1. For how many projects have you consulted an architect? Only 1 [ ] 2-5 [ ] 5-10[ ] More than10 [ ]
2. When you consulted an architect, which of the following best describe your status?

Private individual client [ ] Private Organisation Client [ ] Representative of Public Organisation [ ]

1. For which project did you engage an architect? Personal House [ ] Rental Apartment [ ]

Office Building [ ] Educational Building [ ] Religious building [ ] Industrial Building [ ] Healthcare building [ ] Entertainment Building [ ]

1. Which type of service did you procure from the architect? :

Design only [ ] Construction only [ ] Design and Construction [ ] Project management [ ]

Interior design [ ] Renovation and installation [ ] Others (please specify) ....................................

1. How did you get to know about the architect: Advertisement [ ] Referral[ ] Relative[ ]
2. How would you rate your overall satisfaction with the architectural services that you received

Not satisfied at all [ ] Not satisfied [ ] Undecided [ ] Satisfied [ ] Highly satisfied [ ]

1. How likely is it that you would contact your last architect for the next project?

Very Unlikely [ ] Unlikely [ ] Undecided [ ] Likely [ ] Very likely [ ]

1. What is the tendency that you would recommend your last architect to you friends and associates?

Very Unlikely [ ] Unlikely [ ] Undecided [ ] Likely [ ] Very likely [ ]

1. Please rate the quality of the service tour received.

Very Low quality [ ] Low quality [ ] Neutral [ ] High Quality [ ] Very High Quality [ ]

1. Please rate the value of the service you received compared for the cost of the project

Cost far outweighed value of service [ ] Cost matched the value of service [ ]

Value of the service is greater than the cost [ ]

**SECTION B. CRITERIA USED BY CLIENTS FOR THE SELECTION OF ARCHITECTS**

1. To what extent did the following influence your selection of architect?

|  |  | Not influential at all | Not influential | Undecided | Influential | Highly influential |
| --- | --- | --- | --- | --- | --- | --- |
|  | Geographical location |  |  |  |  |  |
|  | Reputation |  |  |  |  |  |
|  | Experience |  |  |  |  |  |
|  | Expertise in design of particular building types |  |  |  |  |  |
|  | Service Reliability |  |  |  |  |  |
|  | IT proficiency |  |  |  |  |  |
|  | Convenience |  |  |  |  |  |
|  | Personal Relationship |  |  |  |  |  |
|  | Ease of communication |  |  |  |  |  |
|  | Availability |  |  |  |  |  |
|  | Past relationship |  |  |  |  |  |
|  | Financial Consideration |  |  |  |  |  |
|  | International scope of architect |  |  |  |  |  |
|  | Customer service |  |  |  |  |  |
|  | Quality of previous service |  |  |  |  |  |
|  | Value added services |  |  |  |  |  |
|  | Accessibility of architect in urgency |  |  |  |  |  |
|  | Religious affinity |  |  |  |  |  |
|  | Ethnic affinity |  |  |  |  |  |
|  | Competence/ professionalism |  |  |  |  |  |
|  | Friendliness |  |  |  |  |  |
|  | Recommendation |  |  |  |  |  |
|  | Professional Advise |  |  |  |  |  |
|  | Patience and help  Established relationship |  |  |  |  |  |

**SECTION C: RESPONDENTS’ SATISFACTION WITH THE ARCHITECTURAL SERVICES**

1. Please rate your level of satisfaction with the following aspects of the services

| **S/N** |  | **Very Satisfied** | **Satisfied** | **Neutral** | **Dissatisfied** | **Very Dissatisfied** |
| --- | --- | --- | --- | --- | --- | --- |
|  | Adequate consultant experience |  |  |  |  |  |
|  | Proper methods in rendering service |  |  |  |  |  |
|  | Proper coordination of resources |  |  |  |  |  |
|  | Construction and supervision |  |  |  |  |  |
|  | Effective communication |  |  |  |  |  |
|  | Decision making |  |  |  |  |  |
|  | Attainment of design requirement |  |  |  |  |  |
| **S/N** |  | **Very Satisfied** | **Satisfied** | **Neutral** | **Dissatisfied** | **Very Dissatisfied** |
|  | Cost estimates |  |  |  |  |  |
|  | Labour productivity |  |  |  |  |  |
|  | Waste reduction/ management |  |  |  |  |  |
|  | Effective control of budget |  |  |  |  |  |
|  | Display of expertise |  |  |  |  |  |
|  | Speed of service |  |  |  |  |  |

**SECTION D: FACTORS THAT INFLUENCE CLIENTS’ SATISFACTION WITH ARCHITECTURAL SERVICES**

1. Please indicate your level of agreement with the following:

| **S/N** | **Factors** | **Strongly**  **agree** | **Agree** | **Disagree** | **Strongly**  **disagree** | **Not Sure** |
| --- | --- | --- | --- | --- | --- | --- |
|  | The architect understood the kind of help I wanted |  |  |  |  |  |
|  | The architect seemed to have a different idea about my project objective |  |  |  |  |  |
|  | The architect is always available when I want to discuss |  |  |  |  |  |
|  | The architect informed me about decisions made on my behalf |  |  |  |  |  |
|  | I received the type of service I was looking for |  |  |  |  |  |
|  | I like the way the architect relates with me |  |  |  |  |  |
|  | The architect was often too busy to attend to my requests |  |  |  |  |  |
|  | The architect always properly handled problems that arose during the course of the project |  |  |  |  |  |
|  | The architect displayed adequate knowledge about architecture |  |  |  |  |  |
|  | The architect always answered my questions satisfactorily |  |  |  |  |  |
|  | The architect understood my specific needs |  |  |  |  |  |
|  | The architect rendered quality service. |  |  |  |  |  |
|  | The charges were reasonable |  |  |  |  |  |
|  | The architect follows through on his promises |  |  |  |  |  |
|  | The architect did things right the first time |  |  |  |  |  |
|  | The architect was friendly |  |  |  |  |  |
|  | The architect provided easy access to needed information |  |  |  |  |  |
|  | The architect’s office was welcoming |  |  |  |  |  |
|  | The architect was always willing to help |  |  |  |  |  |
|  | The architect was caring and concerned |  |  |  |  |  |
|  | The architect was prompt at attending to my requests |  |  |  |  |  |
|  | The architect displayed competence |  |  |  |  |  |
|  | The architect gave me personal attention |  |  |  |  |  |
|  | The architect was consistently courteous |  |  |  |  |  |
|  | The architect maintained professionalism |  |  |  |  |  |
|  | The architect kept my dealings confidential |  |  |  |  |  |
|  | The architect met my expectations |  |  |  |  |  |
|  | The architect was dependable in handling service problems |  |  |  |  |  |
|  | The architect provided services at promised time |  |  |  |  |  |
|  | The architect explained the process well |  |  |  |  |  |
